# Gaps in vaccine management practices during vaccination outreach sessions in rural settings in southwestern Uganda

**DOI:** 10.1186/s12879-023-08776-x

**Published:** 2023-11-03

**Authors:** Atwiine Flavia, Bagenda Fred, Turyakira Eleanor

**Affiliations:** Department of Community Health, Faculty of Medicine, Mbarara University of Science & Technology, Mbarara, P.O. BOX 1410, Mbarara, Uganda

**Keywords:** Health care providers, Outreach immunisation, Key informants, Vaccine management, Socio-ecologic framework

## Abstract

**Background:**

Outreach efforts were developed to bolster people’s access to and use of immunization services in underserved populations. However, there have been multiple outbreaks of diseases like measles in Uganda, prompting policy makers and stakeholders to ask many unanswered questions. This research study was created to uncover the discrepancies between vaccine management practices at immunization outreach sessions in rural South Western Uganda compared with existing standards.

**Methods:**

The observational qualitative study, was done in 16 public health facilities across four districts of Uganda. Data were collected using in-depth interviews, facility record reviews, and observation. We assessed the vaccine management procedures before immunization session, transportation used, set up at the outreach site, management practices during the outreach session and packing of vaccines - according to World Health Organization immunization practice recommendations. The data were transcribed, coded and categories were formed and triangulated. Themes were generated based on a socio-ecologic framework to gain a better understanding of healthcare provider practices during immunization sessions.

**Results:**

Fifty-one individuals were interviewed; four Assistant District Health Officers, four cold chain technicians, 15 focal persons for the Expanded Program on Immunization, and 28 health care providers. The respondents’ mean age was 35, 43 (84.3%) were females and 24 (47.1%) had a diploma. 11 (69%) outreaches were conducted at a distance of 5-12 km from the health facility and 7 (44%) were conducted in a building. For 8 outreaches (50%) health facility staff did not check the vaccine vial monitor status before the outreach while 12(75%) did not keep the vaccine hard lid cover closed during the sessions. The main areas of concern were insufficient vaccine integrity monitoring, improper handling and storage practices, deficient documentation, and inadequate vaccine transportation. These were similar across immunization outreach sites regardless of vaccine preventable disease outbreaks occurrences. The majority of these gaps were located at the individual level but were enabled by policy/environmental factors.

**Conclusions:**

There are poor vaccine management procedures during outreach sessions contrary to established guidelines. Specific tactics to tackle knowledge deficiencies, health worker attitude, and fewer equipment shortages could improve compliance to guidelines.

## Background

Vaccination is one of the most cost-effective and successful public health interventions for protecting people, both adults and children under five years, from vaccine preventable diseases [1–3]. Childhood vaccination has contributed to major global reductions in morbidity and mortality due to infectious diseases, preventing more than 2.5 million child deaths per year [2–4]. As of 2018, the total world population of children < 5 years of age was roughly estimated at 679 million. Of these children, an estimated 5.3 million died of all causes in 2018, with an estimated 700,000 who died of vaccine-preventable infectious diseases; 99% of the children who died had lived in low and middle-income countries [5]. Vaccination is a fundamental intervention towards attaining Sustainable Development Goal (SDG) target 3.2, which aims at reduction of under-five mortality to less than 25 per 1000 live births by 2030 [4, 6].

Many strategies have been used to improve access and utilization of routine childhood vaccination services; one of which is establishing outreach vaccination services for populations which are hard to reach and limited access to facility-based services [3, 7]. Outreaches are planned, regular and periodic single-day visits by qualified staff from a health facility to populations located 5–15 km from the facility [3]. Outreaches often play an important role in systematically delivering vaccination services to a large proportion of the population, in some cases reaching more than 50% of the target population [8].

The routine child immunisation program in Uganda aims to deliver an outright number of antigens in a timely, safe, and potent way to all children and women [9, 10]. More than 50% of children in rural communities in Uganda access vaccination services mostly through outreaches in remote settings at least five kilometres away from health facilities [11, 12]. High coverage, availability of potent vaccines, and timely delivery of scheduled immunizations are key to achieving the full benefits of vaccination [13, 14].

It is known that effective vaccine storage, handling, and transportation are key components of immunization programs because any loss of potency in a vaccine is permanent and irreversible [15]. It is also known that vaccines respond differently to heat, light and freezing which makes adherence to cold chain recommendations a critical component to maintaining quality and efficacy of these products [14]. The World Health Organisation recommends vaccines be stored and kept at temperatures between + 2 °C and + 8 °C to remain safe and potent [7]. Inconsistent adherence to vaccine management guidelines might leave some communities vulnerable to outbreaks of vaccine preventable diseases and result in economic losses when vaccine potency is affected by compromised cold chain [16, 17].

Despite increased outreach immunization coverage in Uganda’s hard to reach areas, several outbreaks of vaccine preventable diseases like measles have been reported over time leaving a lot of unanswered questions with policy makers and other stakeholders [2]. Plausible explanations for the emergence of vaccine preventable diseases in vaccinated populations include low completion rates and compromised potency of the vaccines. This is further supported by the World Health Organization which noted that 25% of all vaccine products reach their destination in a degraded state [18]. Most research concerning adherence to vaccine management guidelines have focused on practices within the health facility, yet more than 50% of children in Uganda are vaccinated during outreaches. This study looked at gaps in vaccine management practices at outreach vaccination sessions in rural Uganda.

## Methods

An observational descriptive study was conducted using qualitative methods. These included individual face-to-face in-depth semi-structured interviews with health care providers involved in vaccination outreaches, key informant interviews, and observation using a checklist adapted from the World Health Organisation practical guide of conducting outreaches. This checklist was used to assess vaccine management procedures at the health facility during preparation for immunisation outreach, transport means used, set up at the outreach site, vaccine management practices during the outreach session and packing of leftover vaccines. The key informants included EPI focal persons, district cold chain technicians and Assistant District Health Officers in charge of Maternal and Child Health. Interview guides had a preset list of open-ended questions and data collectors could probe for clarity.

This study sought to find out the gaps in vaccine management practices during vaccination outreach sessions in rural settings in Southwestern Uganda so as to identify opportunities for improving the immunisation program. Gaps in vaccine management were identified if healthcare providers executed procedures in ways that were contrary to the recommended WHO vaccine management practices. These included but were not limited to vaccine cold chain management, staffing and training, vaccine storage and temperature monitoring, vaccine inventory management, vaccine preparation and transport. Data were collected from four (4) purposively selected districts of Kasese, Mitooma, Rubirizi and Rwampara in South Western Uganda. Kasese and Rubirizi had registered measles and Rubella outbreaks in 2019, while Mitooma and Rwampara had not registered any outbreaks of any vaccine preventable diseases during the same period. Using the district Reach Every District/Reach Every Child (RED/REC) categorization, in each district, two (2) health facilities which had registered low vaccination coverage for vaccine preventable disease outbreak [identified by using the Health Management Information System (HMIS) tool] in 3 years preceding the study and two health facilities which had maintained a high vaccination coverage were studied. Two (2) eligible health care providers were purposively selected and interviewed at each selected health facility. However, 28 and not 32 interviews (as planned) were conducted with the health care providers because certain health facilities had only 1 health care provider and 1 EPI focal person involved in outreach immunisation sessions. Key informants were purposively selected based on their relevant experience in vaccine management which enabled them to share their perspectives on factors influencing vaccine management practices and their opinions on future directions for improving vaccine management. 23 key informant interviews were conducted. Key informants included EPI focal persons (15) instead of the 16 because 1 health facility, its cold chain technician also worked as the EPI focal person; district cold chain technicians (4) and Assistant District Health Officers in charge of Maternal and Child Health (4). All key informants were asked to schedule their most convenient time for the interviews without disrupting their duty activities and were interviewed from their respective offices. None of the potential participants refused to participate or later withdrew their consent. Each interview lasted for approximately 45 min to 1 h. Data saturation had been reached by the time all interviews were completed and no interviews were repeated.

Data were collected by the first author and two trained research assistants who were both Bachelors degree holders in Nursing who were known not to have any work-related relationship with the participants to prevent any biased responses. Data collectors had Good Clinical Practice training as well as experience in conducting health research. Training of research assistants was conducted for 3 days to ensure detailed understanding of the study objectives, study tools, maintenance of confidentiality and the entire research process. Study tools were pretested in a level 4 health centre of a similar setting as the study area to check for accuracy and consistency and improve validity before the data collection process. Prior to the interviews, potential participants interested in the study were informed of the risks and benefits associated with participation and provided written informed consent. They were assured that the study would not in any way be used as a means of evaluating their performance, that any information they gave would be kept confidential and their individual names would not be revealed in publications. Each participating health facility and respondents were assigned unique identifiers. This informational and trust-building process made the participants relaxed and comfortable conducting immunisation outreach procedures as they always did.

All interviews were audio recorded and field notes taken as a backup of the audios and to capture any information that may have been missed during the interviews. All Audios were transcribed immediately within a period of 2 weeks after data collection and stored on a password protected computer. No transcripts were returned to participants for comments or correction.

In the analysis, field observation notes and interview transcripts were reviewed multiple times. Thematic analysis, which entails searching across a data set to identify, analyse, and report repeated patterns was used. It is a method for describing data, but it also involves interpretation in the processes of selecting codes and constructing themes [19]. The themes created were categorised using the socio-ecological framework to identify gaps in vaccine management during immunisation outreach sessions at individual, interpersonal, community/organizational (health facility), and policy/enabling environment levels. Data coding, category formation and themes generation were done to enable a better understanding of health care providers’ vaccine management practices contrary to the WHO guidelines. Data captured by study observation checklists were entered in Microsoft Excel software and analysed in relation to the WHO immunization practice recommendations. Findings from analysis of interview transcripts, field observation notes, and vaccine management checklist data were triangulated.

### Overview of WHO recommendations for vaccine management during outreaches

During the outreach vaccination session, the cold box should be placed in a shade and a foam pad used to hold opened vials at the top of the vaccine carrier and keep the lid cover tightly covered at all possible times [20]. The health care provider then reviews clients’ immunisation cards to determine the eligible vaccinations based on clients’ age, and possible contraindications. A contraindication is a health condition in the recipient that increases the likelihood of a serious adverse reaction to a vaccine. Some of these contraindications include severe allergic reaction, severe immunosuppression, history of intussusception, Encephalopathy etc. Healthcare provider should reconstitute all vaccines with their matched diluents, administer these vaccines using the recommended techniques and injection sites, and discard the used needles in the safety box. All vaccines should be recorded in the register, tally sheet and immunisation cards. The health care provider should communicate the next visit date and the potential adverse events following immunisation (AEFIs). After the immunisation session, all opened vials that do not contain a preservative should be discarded, vaccine vial monitor checked for the remaining vaccines, and date of opening on the multi-dose vaccine vials recorded. Outreach immunisation session summary reports should be completed and dates for the next outreach communicated. These summary reports are then compiled to monitor progress of immunisation at the health facility and provide information to devise means of improvement where needed.

## Results

### Participant characteristics

Data were collected from 16 health facilities including five health centre IVs, and 11 health centre IIIs, in four districts of southwestern Uganda. A total of 51 participants were interviewed including four Assistant District Health Officers (ADHO-MCH) in charge of maternal and child health service coordination and monitoring in each district, four cold chain technicians, 15 Expanded Program on Immunisation (EPI) health centre focal persons and 28 health care providers. The role of ADHOs in charge of maternal and child health is to assist the District Health Officer in ensuring efficient, effective and affordable delivery of Maternal Child Health and Nursing Services for the wellbeing of the population of the District and ensure quality assurance in all Health Institutions in the District. The number of interviews conducted with the health care providers were 28 and not 32 as planned because certain health facilities had only one (1) health care provider and one (1) EPI focal person involved in outreach immunisation sessions. The enrolled participants had a mean age of 35 (29, 42) years, 43 (84.3%) were females, 24 (47.1%) had a diploma as their highest level of education, 16 (57%) were midwives. They had a median duration of 9 years of professional experience and 5 years’ experience in vaccine management (Table 1).

**Table 1:** Participant and outreach site characteristics

Variable	n(%)
Age in years: median(IQR)	35(29, 42)
*Gender*	
Females	43(84.3)
Males	8(15.7)
*Highest level of education*	
Degree	4(7.8)
Diploma	24(47.1)
Certificate	23(45.1)
Years of professional experience: median(IQR)	9(3, 18)
Duration of experience in vaccine management: median(IQR)	5(2, 14)
**Health care provider’s training/ cadre (n = 28)**	
Midwife	16 (57%)
Nurse (comprehensive, enrolled, registered)	5 (18%)
Nursing Assistant	3 (11%)
Vaccinator	1 (4%)
Counsellor	1 (4%)
Medical Entomology	1 (4%)
Laboratory Technician	1 (4%)
Distance to the outreach site (n = 16)	
2–4 km	5 (31%)
5–12 km	11 (69%)
Nature of the outreach site (n = 16)	
In a building	7 (44%)
Under tree	5 (31%)
Tent/ veranda	4 (25%)

IQR: Inter-quartile range

### Nature of outreach sites

Some health facilities were conducting one outreach in a week, others one outreach in 2 weeks and some, one outreach per month. Only one outreach session per health facility was attended by the study team. Nine of the 16 outreach vaccination sessions observed were conducted in the open; under the tree (5 of 16), or veranda (4 of 16) (Table 1). Where outreach sessions were conducted in the open, there were no gazetted buildings for community vaccination activities thus health care providers always improvised. 11 outreaches were conducted at a distance of 5-12 km from the health facility. It was noted that there were no differences in the gaps identified in vaccine management among health facilities with less frequent outreaches compared to those that were conducting outreaches more often.

### Observed gaps in vaccine management practices

The gaps in vaccine management practices during vaccination outreach sessions were categorized into themes at the different levels of individual, interpersonal, community/health facility and policy or enabling environment levels in line with the socioecological framework. These gaps were concerned with the cold chain and included insufficient monitoring of vaccine integrity, handling and storage affecting vaccine quality, poor documentation, refrigerator management, refrigerator overload, transportation of vaccines and conducting the outreach in inappropriate spaces.

### Individual level

#### Theme 1: insufficient monitoring of vaccine integrity

***Sub theme 1: Inability to use the vaccine vial monitor***.

Vaccines that require reconstitution come with vaccine vial monitors to enable health care providers determine whether the vaccine to be administered has been exposed to heat or not by observing the colour change on the vaccine vial monitor (VVM). The majority of health care providers reported not knowing how to check the vaccine vial monitor (VVM) and some did not know what it was.

*“When it is either stage 1, you give. Stage 2 and 3…Ahhh am not sure about those things but we just see there and we determine that this thing can be given or not***”.** Health care provider, RW01- 01.

It was also observed that at 8 of 16 health facilities, health care providers did not check the vaccine vial monitor status while preparing for the outreach **(**Table 2**).**

**Table 2:** Key observations made in the 16 health facilities using the observation checklist

Variable	Frequency n = 16(%)
*Health facility level*	
Lack electronic freeze indicator	15 (94%)
Lack foam pads or not enough	3 (19%)
*Health worker- Individual level practices*	
Did not check for open vial dates on the multi-dose vaccines	13 (81%)
**Did not check the vaccine vial monitor status**	**8 (50%)**
Did not properly place vaccines, diluents and correct number of ice packs in the vaccine carrier	1 (6%)
**Did not keep the vaccine hard lid cover closed tightly**	**12 (75%)**
Did not administer each vaccine according to the recommended technique and correct injection site	5 (31%)
Did not communicate key messages including potential AEFIs and date of next visit	7 (44%)
Did not discard all reconstituted vaccines and liquid multi-dose vaccines	4 (25%)
Did not check vaccine vial monitor status for vaccines containing preservatives before returning them to the refrigerator	9 (56%)
Did not record dates of opening on vials that could be used and didn’t place them in the ‘first box’ in the refrigerator when back to the facility	12 (75%)
Did not complete session summary reports	7 (44%)

#### Sub theme 2: Failure to check expiry dates

Failure to check expiry dates was noticed in all outreach sites. None of the health care providers would check the expiry dates on the vaccines and this was admitted by most health care providers claiming that it is the work of the EPI focal person.

*“Like sometimes, they find a staff has gone to mix like BCG and doesn’t check on the manufacturer or the expiry date. Though I know that in my store, I have the update… expiry dates which are updated, but you find he/she doesn’t want to check”.* Key informant, M02- 01.

### Theme 2: poor handling and storage

Poor handling of vaccines manifested in the forms of holding the vaccine vails incorrectly and opening multiple vaccine vails at once during outreach immunisation sessions. Improper storage practices included using few or unconditioned ice packs, keeping vaccine carriers open throughout the outreach vaccination session and returning vaccines to the refrigerator that should be discarded. Although less common, the carrying vaccines in inappropriate material like a safety box was also observed.

#### Sub theme 1: poor storage of vaccines

Proper vaccine storage is very important to maintain the potency of the vaccines. However, some health care providers admitted to be storing these vaccines and their diluents poorly during outreach sessions. While vaccines were mostly packed in recommended vaccine carriers for outreach sessions, it was reported in the interviews and also seen in one outreach site that vaccines were carried in a safety box.

“*…like when we are taking the vaccines in the safety box, of course it is not right*…” Health care provider, RW02- 01.

It was observed that some health care providers used few and/or unconditioned ice packs during outreach immunisation sessions. In addition, at 12 of 16 outreach sessions, vaccine carriers were seen open throughout the outreach session instead of being tightly covered when not vaccinating; it was observed that health care providers did not keep the vaccine hard lid cover closed tightly during vaccination (Table 2).

#### Sub theme 2: poor holding of vaccine vials

Vaccine vials are supposed to be held from the neck to minimize contact with the provider’s body to maintain vaccine temperature. During outreach vaccination sessions, it was observed that some health care providers held the vials in their folded palm contrary to the guideline of holding the neck or the tip. In the interviews, health care providers agreed with this field observation reporting that some health care providers touch the vial ‘everywhere’ which can lead to warming.

*“Ehhhhhh (she laughs) of course when you are going to mix the vaccine… You are supposed to touch it in the neck but you find someone is touching it everywhere which can make the vaccine to be warm”.* Health care provider, RW02- 01.

#### Sub theme 3: opening multiple vaccine vials at once

The guidelines encourage health care providers to open one vaccine vial at a time when the clients are there to avoid removing more vaccines from below the sponge in the vaccine carrier while vaccinating to maintain the right vaccine temperatures and avoid wastages. However, it was observed that health care providers had a tendency of reconstituting all vaccines at once even before a reasonable number of clients turned up. This gap was also known to district EPI coordination teams.

*“There are facilities which are still opening more than two vials at a time at the start of the session. Whereby it is not advisable for a person who is going to vaccinate to open more than one vial. To open more than one vial at a time when clients are there”.* Key informant, K-CCT.

#### Sub theme 4: not labelling multi-dose vaccine vials

Vaccine labelling is very crucial in vaccine management. However, it was reported in most outreaches that health care providers were not labelling the multi-dose vaccine vials after outreach immunisation sessions. Even during the outreaches, no health care provider was observed labelling any multi-dose vial.

*“The one they opened yesterday when I had left this place, I found it there this morning. It was not labelled. We say that when you open BCG and measles, after 6 h, it should be discarded. So now, should I have used it or not used it?”*

Health care provider, M01- 01.

The practice of returning partially used multi-dose vaccine vials to the refrigerator irrespective of duration spent outside the fridge was noted. It was observed that vaccines which are meant to be discarded after an outreach immunization session, particularly measles and BCG vaccines, were often returned to the refrigerator. This practice was also reported by health care providers themselves and EPI focal persons at health facilities. In addition to the lack of knowledge, this practice may also be influenced by the late time of returning from the immunization outreach session and poor attitude of health care providers.

“*You can find you are returning it is like at 4pm. You are rushing to go home, your time off work is coming. So, you come and put everything in the fridge, and you go”.* Health care provider, RW02- 01.

### Theme 3: poor documentation

#### Sub theme 1: poor tracking of vaccine usage

In most outreach sites, it was discovered that there was poor documentation of vaccines yet it is known that when documentation is poor, timely requisition and supply of vaccines can be affected, leading to stock outs and wastages. It was observed during the immunisation outreach sessions that none of the health care providers filled the vaccine control book. This observation was further backed up by interview data from healthcare providers themselves and their supervisors. Some supervisors even pointed out how they had challenges in balancing vaccines due to the poor documentation.

*“I can call it a gap in balancing the vaccines. Because you find that someone goes to the outreach but doesn’t show how many they had taken and how many have come back. So you find it hard to first go and ask….how many did you take…then… so we are not doing the documentation….”.* Health care provider, M01- 02.

Despite the gaps identified, there were quite a number of good vaccine management practices observed in all the 16 outreaches including using foam pads while in the outreaches, using auto-disable syringes, reconstituting all vaccines with the matching diluents for the lyophilized vaccines (e.g. measles-rubella), completing immunisation registers and tally sheets, determining eligible vaccinations based on the national schedule and client’s age and immediately discarding all the used syringes in a safety box. Fourteen of 16 health facilities had two or more health care providers involved in the outreach immunisation sessions. At one of the outreach sites, there was only one health care provider who didn’t have even a lay community health worker (Village Health Team-VHT) to support her, and one (1) facility involved a VHT in vaccine management during outreach sessions.

### Interpersonal level

No gaps were identified at this level.

### Policy/enabling environment level

#### Theme 4: vaccine transportation

Transporting vaccines by untrained local boda boda cyclists was reported by district-level health managers who supervise disbursement of vaccines from district vaccine stores (DVS) to health facilities and provide ongoing support regarding issues of cold-chain management.

*“some people just come to pick vaccines without requisitions and usually they send some boda bodas (local motor cyclist)….They send some boda boda men and yet they will not know how to carry the vaccines safely from here to the facility****”.*** Key informant, *R-ADHO.*

#### Theme 5: arrangement of vaccines in the fridge

Mixing of freeze sensitive and heat sensitive vaccines in the same chamber was observed in several health facilities.

*“Maybe some of them are doing them ‘to whom it may concern’. Maybe there is an EPI focal person; is going to come and arrange them with time and most of them….maybe they think the EPI focal person is forever there to arrange for them.”* Key informant, ***R02-01***.

#### Theme 6: vaccine vial monitor (VVM)

Some participants reported that some vaccines come from National Medical Stores and reach District Vaccine Stores when they are already in stage 2.

*“Other vaccines do arrive at districts when they are in stage 2. .... we have 4 stages of vaccine management whereby the 1st and the 2nd are supposed to be used. The 3rd and the 4th, we are supposed to discard vaccines. And sometimes the rota and IPV normally reach here at district level when they are in stage 2. And by the time we deliver them to facilities, sometimes you find other facilities do keep them and the VVM goes off to reach stage 3”.* Key informant, *K-CCT.*

#### Theme 7: refrigerator overload

Overloading the vaccine fridges at the DVS were reported by several cold chain technicians and Assistant District Health Officers in charge of maternal and child health.

“*No funds for transportation of vaccines and when they bring other vaccines, you find that we are overloaded and the fridge is too small”.* Key informant, *RW-CCT.*

## Discussion

We used observational and qualitative methods to identify gaps in vaccine management practices during immunisation outreach sessions in rural settings in southwestern Uganda using the socio ecologic model as the overarching framework. In this study, we found that the main gaps in vaccine management during outreach immunisation sessions were insufficient vaccine integrity monitoring, poor handling and storage, poor documentation and improper vaccine transportation. Regardless of the performance categorization of health facilities, gaps in vaccine management were noted in all health facilities.

### Insufficient vaccine Integrity monitoring

Vigilance in monitoring vaccine integrity with reliable tools is needed to ensure that only vaccines meeting acceptable standards of potency are administered [14]. In this study, we found out that some health care providers did not know how to check the vaccine vial monitor (VVM) status, none of the health care providers checked for the expiry dates of the vaccines while preparing to go for the outreach immunisation sessions. In addition, some health workers did not know how to perform the shake test to check the freezing status of the vaccines.

### Poor vaccine handling and storage

Vaccine management guidelines specify that vials taken for an outreach session, even if not used, do not usually return to the cold chain if vaccine vial monitors (VVM) are not attached [21]. In this study, we observed opened vials (even if not used) discarded at the end of the immunisation session leading to vaccine wastage, an issue that has been previously noted [22]. The underlying behaviour among health workers participating in the current study was that multiple vaccine vials would be opened at the beginning of the outreach immunisation session even before enough clients turned up to consume all the opened vials and this led to vaccine wastage. This is in agreement with a study conducted by Divya et al. that discovered wastage of multi-dose vial of PCV varying from 0–20% [22].

It was also observed that some health care providers would return all remaining vaccines to the fridge after the immunisation outreach sessions irrespective of how long they had spent in the open. It has been reported previously that irregular training of health facility personnel who manage vaccines could limit awareness and competence to consistently follow vaccine management guidelines [23]. Personnel training is a key component of cold chain management [24]. In addition, our study revealed that workload may be a contributing factor to poor practices in the storage of vaccines in refrigerators after completion of outreach immunisation sessions. It has been noted that health care provider shortages lead to higher workload which can affect monitoring of the cold chain and compromise adherence to vaccine management guidelines [25].

### Poor documentation

Our study has shown that health worker failure to label opened multi-dose vaccine vials and to fill vaccine control books during outreach immunisation sessions is still a common problem. Delayed and unreliable documentation of vaccine usage leads to limited use of local data and affects forecasting of future needs [26]. These gaps have been attributed to limited supervision whereby managers rarely review device and vaccine records [12]. These gaps in documentation coupled with limited supervision may lead to vaccine management issues going unreported and unaddressed.

### Improper vaccine transportation

Proper vaccine transportation is very important to maintain the potency of the vaccines. Most immunisation outreach sites in the four districts of western Uganda have rugged and hilly terrain with marram roads that become impassable during the rainy season. This was also identified through a systematic review of low income countries by Partapuri et al. who noted that immunisation outreach posts are located at least 5 km away from the health centre, in communities with poor roads, dusty environments and no electricity [8] requiring prioritization of safety during storage, transportation and delivery of vaccines.

In this study, some health care providers admitted to be transporting vaccines and their diluents poorly both to the health facilities and outreaches to the extent that even one health facility was seen carrying vaccines in a safety box to the outreach site and another health care provider admitted the same too. It was observed that some health care providers used few and or unconditioned ice packs when going for outreach immunisation sessions. Even vaccine carriers were seen open throughout the outreach session instead of being tightly covered when not immunizing.

### Strengths and limitations

This qualitative study provided a deeper understanding of vaccine management practices during vaccination outreach sessions in rural settings in southwestern Uganda and hence identified the gaps that could be addressed. This study is qualitative by design and the study sample being composed of only 4 districts in southwestern Uganda is not necessarily representative of all districts in southwestern Uganda and the entire country. However, triangulation of study results using several data collection tools was done and findings from this study have been described in detail with supporting quotes to enable transferability to similar contexts and settings.

## Conclusion

The study revealed poor vaccine management practices during outreach vaccination sessions contrary to guidelines for health workers. Most of the gaps in vaccine management during vaccination outreach sessions were identified at the individual health worker level. Regardless of the categorization of the study districts and health facilities, the gaps in vaccine management practices during vaccination outreaches were noted to be generally similar across vaccination outreach sites, health facilities and districts.

### Recommendations

Specific strategies to address knowledge gaps in vaccine monitoring, appropriate storage and handling; health worker teamwork building; as well as addressing small equipment shortages, could tremendously improve adherence to vaccine management guidelines during outreach immunisation sessions. Health facility managers, district leaders, the Uganda Ministry of Health and implementing partners like Gavi, the Vaccine Alliance should support regular and continuing education on appropriate vaccine management practices.

## Data Availability

All data generated or analysed during this study are included in this published article. There are no restrictions to data sources, however, details of the full data may be accessed through the corresponding author; Ms. Atwiine Flavia, Email: fatwiine@must.ac.ug.

## Abbreviations

AEFI: Adverse events following immunisation
BCG: bacille Calmette-Guérin
CCT: Cold Chain Technician
EPI: Expanded program on Immunisation
GAVI: Global Alliance for Vaccines and Immunisation
KI: Key informant
VHT: Village Health Team
VVM: Vaccine Vial Monitor
WHO: World Health Organisation
